# The Protective Effect and Molecular Mechanism of Tetrandrine on Male Reproductive Damage Caused by Silicon Dioxide

**DOI:** 10.3390/toxics14010087

**Published:** 2026-01-18

**Authors:** Hong-Mei Li, Xiao-Qi Zeng, Qing Chang, Yu-Xin Sheng, Ya-Jia Pu, Yi Wang, Bin Cheng, Hong-Hui Li, Jie Xuan, Ling Zhang, Hai-Ming Xu

**Affiliations:** 1 The Key Laboratory of Fertility Preservation and Maintenance of the Ministry of Education, School of Basic Medicine, Ningxia Medical University, Yinchuan 750004, China; lihongmei@nxmu.edu.cn (H.-M.L.); 240230020231@nxmu.edu.cn (X.-Q.Z.); seryin55@outlook.com (Q.C.); 230230020186@nxmu.edu.cn (Y.-X.S.); 240230020194@nxmu.edu.cn (Y.-J.P.); wangyi1025leibo@163.com (Y.W.); chengbin224@163.com (B.C.); nxmulhh@nxmu.edu.cn (H.-H.L.); 2 The Key Laboratory of Environmental Factors and Chronic Disease Control, School of Public Health, Ningxia Medical University, Yinchuan 750004, China; 3 The Fifth People’s Hospital of the Ningxia Hui Autonomous Region, Shizuishan 753000, China; xuanjie975@163.com

**Keywords:** silicon dioxide, male reproductive damage, tetrandrine, antagonistic effect, molecular mechanism

## Abstract

The long-term inhalation of free silica dust causes silicosis—a prevalent occupational hazard—yet its systemic effect on male reproductive toxicity remains underexplored. Tetrandrine (Tet) is the only plant-derived anti-silicosis drug approved in China. This study investigates silica (SiO_2_) -induced male reproductive damage and evaluates Tet’s protective effects. Sixty male C57BL/6 mice (6–8 weeks) were divided into control, SiO_2_ exposure, and SiO_2_ + Tet groups. SiO_2_ was administered via intranasal infusion and Tet via gavage. Mice were sacrificed at day 7 (male reproductive injury model corresponding to the pulmonary inflammation stage) and day 42 (male reproductive injury model corresponding to the pulmonary fibrosis stage). Analyses included sperm morphology, testicular transcriptome sequencing, RT-qPCR, and immunofluorescence. At day 7, SiO_2_ exposure upregulated testicular inflammatory markers, which were partially mitigated by Tet. At day 42, SiO_2_ increased sperm deformity and testicular fibrosis markers (fibronectin and vimentin); Tet intervention reduced these abnormalities. Transcriptome analysis revealed distinct gene expression patterns at day 7 versus day 42, indicating time-dependent injury mechanisms. Tetrandrine alleviates silica-induced reproductive damage in male mice, suggesting potential therapeutic applications for occupational silica exposure and expanding the understanding of silica toxicity beyond the respiratory system.

## 1. Introduction

Silicon dioxide (SiO_2_), as the main component of the Earth’s crust, exists widely in both amorphous and crystalline forms [1,2]. Since 1997, the International Agency for Research on Cancer (IARC) has classified crystalline silica (in the form of quartz/cristobalite) as a Group 1 human carcinogen. The risk of lung cancer caused by occupational exposure to it continues to pose a major public health threat in the global mining, construction and artificial stone processing industries [3,4]. According to epidemiological investigations, approximately 23 million occupational workers in China have been exposed to silica dust for a long time [5]. Inhalable particles with a diameter less than 10 µm can remain in the nasal cavity or be inhaled through the mouth to reach the bronchi, while particles with a diameter less than 2.5 µm can reach the alveoli, inducing irreversible occupational diseases such as silicosis and occupational tumors [6,7]. At the same time, silica inhalation exposure also poses potential threats to other systems of the body (including but not limited to the reproductive system). Recent studies have found that nano-sized silica particles (SiNPs) are widely distributed in the atmosphere due to industrial dust [8]. They have smaller particle sizes and stronger permeability, and can break through the blood–testis barrier and accumulate in testicular tissue. This leads to the apoptosis of spermatogenic cells, a decline in sperm quality, and a decrease in serum testosterone level [9,10,11]. The quantitative analysis results of the literature show that the existing research mostly focuses on the reproductive toxicity of SiNPs. The damage mechanism and protective strategies of traditional inhalable silica dust to the male reproductive system remain unclear, which seriously restricts the reproductive health protection of occupationally exposed populations.

Tetrandrine (Tet) is a dibenzylisoquinoline alkaloid extracted from the root powder of *Stephania tetrandra S*. Moore, a plant belonging to the Menispermaceae family. It is the only approved plant-based anti-silicosis drug in China and has been used in clinical practice since the 1980s [12,13,14]. Its pharmacological effects involve calcium channel blocking and oxidative stress regulation, and it can induce the apoptosis of cancer cells by inhibiting the ROS/JNK signaling pathway [15,16]. It is notable that the potential protective effect of Tet on male reproductive function has begun to emerge. Studies have shown that Tet alleviates erectile dysfunction by inhibiting the Ca^2+^ signaling pathway and improving the diastolic function of corpus cavernosa smooth muscle [17]. This discovery suggests that Tet may exert a protective effect on the male reproductive system by regulating calcium homeostasis and oxidative stress. However, current research on Tet mainly focuses on the fields of anti-cancer or respiratory diseases. Its role in the protection against testicular injury has not been systematically explored, with a particular lack of studies on the intervention of reproductive toxicity caused by traditional inhalable silica exposure, which is one of the key research directions of public health and preventive medicine.

Based on this, this study focuses on the scientific issue of male reproductive damage caused by inhalable silica exposure that is widespread in occupational environments, and at the same time explores the antagonistic effect of Tet on male reproductive damage caused by silica. The research results will expand our understanding of the bodily damage caused by silica exposure in occupational environments, and at the same time provide new insights for the clinical treatment of male reproductive damage caused by silica using Tet.

## 2. Materials and Methods

### 2.1. Materials

#### Test Compounds and Main Reagents and Consumables

Crystalline silica (Sigma-Aldrich, St. Louis, MO, USA; product number: S5631, 80% particle size 1–5 μm, purity 99%) and Tetrandrine (Shanghai Ronghe Pharmaceutical Technology Development Co., Ltd., Shanghai, China; product number: 518-34-3, purity: 99.36%) were used. The relevant information on the main reagents used in this study is shown in Table 1.

### 2.2. Method

#### 2.2.1. Experimental Animals

Sixty SPF-grade male C57BL/6 mice (23 ± 2 g), 6–8 weeks old, provided by the Laboratory Animal Center of Ningxia Medical University (License Number of the Animal Experiment Center: IACUC-NYLAC-20220219) were used. The mice were exposed to a suitable temperature (24 ± 1 °C), humidity (55% ± 15%), and a light cycle of 12 h:12 h, freely feeding and drinking. The feed and bedding were provided by the experimental animal center. All procedures for this experiment were approved by the Medical Ethics Review Committee of Ningxia Medical University (Approval No.: 22023-2760).

#### 2.2.2. Experimental Grouping and Model Establishment

Sixty 6–8-week-old SPF-grade male C57BL/6 mice were adaptively fed for 7 days and then randomly assigned to three groups, the control group, the SiO_2_ model group, and the Tet + SiO_2_ group, with 20 mice in each group. Since this study focuses on the secondary male reproductive damage effect after lung injury caused by SiO_2_ exposure, the modeling method refers to the previous study of our research group [18]. After one week of adaptive rearing of mice, researchers who did not conduct subsequent experiments stratified the mice by body weight according to a random number table and randomly assigned them to the control group, the SiO_2_ group, and the SiO_2_ + Tet group. The specific methods are as follows: Mice in the control group were given the corresponding volume of normal saline by nasal drops and the corresponding volume of corn oil by gavage (calculated based on the weight of the mice, ensuring that the volume gavage for each mouse was proportional to its weight and 40 µL of SiO_2_ was chosen for the nasal drip experiment). The volume calculation method for the control group and the treatment group was the same. Mice in the SiO_2_ model group were given 80 mg/kg SiO_2_ (prepared with normal saline) by nasal drops (nasal drip operation was performed after mild inhalation isoflurane anesthesia was administered to mice) and the corresponding volume of corn oil by gavage. Mice in the Tet + SiO_2_ intervention group were given 80 mg/kg SiO_2_ by nasal drops and 20 mg/kg Tet (prepared with corn oil) by gavage. For the male reproductive injury model corresponding to the pulmonary inflammation phase (abbreviated as D7 model), the following steps were undertaken: from day 1 to day 7, nasal drops and intragastric administration were performed every day. After the experiment, isoflurane inhalation anesthesia was administered and then anatomical samples were collected. For the male reproductive injury model corresponding to the pulmonary fibrosis stage (abbreviated as D42 model), the following instructions were followed: daily nasal drip and gavage operations from D1 to D16; D17-D42 should be administered by gavage every other day. After the experiment, the mice were anesthetized by inhalation with isoflurane, and then euthanized by cervical dislocation. Samples were immediately collected afterwards. In this study, mice were randomly assigned to three treatment groups (Control, SiO_2_, and SiO_2_ + Tet), with 20 animals per group. Sample collection was performed at two time points: day 7 (D7) and day 42 (D42). At each time point, animals were randomly selected for molecular or reproductive analyses as follows. Molecular analyses (testis): For transcriptomic and gene expression analyses, left testicles from 9 mice per group were collected at each time point. These samples were pooled (3 testicles per pool), yielding three biological replicates per group for RNA-seq and RT-qPCR. In addition, the right testicles from 3 of the mice per group were collected for RT-qPCR and immunofluorescence (IF) analysis. Sperm quality analysis: At D42, epididymides from 3 mice per group were also collected for detailed sperm morphology analysis. At the same time, extra animals were included in each group to account for potential losses during the experimental period and were not necessarily used for endpoint analyses.

In this study, to minimize the potential confounding effects of treatment order, measurement sequence, and cage location, complete randomization grouping (to ensure baseline balance between groups), sequential control (Latin square design to balance treatment order), and systematic rotation of cage positions (to mitigate environmental bias) were used. These strategies ensured a uniform distribution of non-experimental factors and reduced systematic bias, with all procedures adhering to international guidelines for laboratory animal welfare and experimental design standards.

During the exposure experiment, the experimenters closely monitored animal responses and adjusted operational procedures promptly based on the animals’ condition. If signs of pain, dyspnea, or other abnormalities were observed, the experiment was suspended immediately, and necessary measures were taken in accordance with ethical guidelines.

The inclusion criteria for the animals in this study were as follows: healthy SPF-grade male C57BL/6 mice aged 6–8 weeks and weighing 23 ± 2 g. Exclusion criteria include individuals who experienced severe illness, accidental death, or abnormally significant weight loss (such as more than 20% of the initial weight) during adaptive feeding or during the experiment. During the data analysis stage, all animals that successfully completed the exposure experiment and obtained valid data were included in the analysis. All the above standards were pre-established in the research protocol before the start of the study.

In this study, all randomly grouped animals successfully completed the entire exposure experiment process without any accidental deaths or sample losses. All the collected sample data were valid. Therefore, all the animals and their data of all experimental groups (control group, SiO_2_ model group, SiO_2_ + Tet treatment group) at time points D7 and D42 were included in the final statistical analysis, and there were no exclusions.

#### 2.2.3. Transcriptome Sequencing

Beijing Youji Technology Co., Ltd. (Beijing, China) was commissioned to conduct transcriptome sequencing and bioinformatics analysis of mouse testicular tissue. A sample merging strategy was adopted, in which tissues from each group were pooled into three independent biological replicates for sequencing (*n* = 3 represents three pooled samples, each composed of equal amounts of tissues from three animals). This approach aimed to enrich and detect group-level consistent expression signals driven by the experimental treatment. Differentially expressed genes (DEGs) were screened using FDR-adjusted *p*-values, which effectively balances false positive control with the retention of true signals. Detailed information on transcriptome sequencing is provided in the Supplementary Materials.

#### 2.2.4. RT-qPCR

Based on the sample merging strategy, we mixed the total RNA of each group of testicular tissues in equal amounts to construct an independent cDNA pool for subsequent analysis. Specifically, after RNA was isolated using the total RNA extraction kit, it was mixed according to the above strategy and the cDNA pool was synthesized using the FastKing gDNA Dismising RT SuperMix kit. Gene expression was determined using RT-qPCR, using these cDNA pools as templates. Amplification and detection were performed using the 2× Universal SYBR Green Fast qPCR Mix kit on the StepOne Plus Real-Time PCR System (Thermo Fisher Scientific, Waltham, MA, USA). Three technical repeat wells were set up for each cDNA cell. G*APDH* was used as the internal reference gene, and the relative gene expression levels between cells were calculated by the 2^−ΔΔCt^ method. The relevant information on the primers is shown in Table 2 and Table 3.

At room temperature, the dewaxed testicular sections were blocked with 5% goat serum for 30 min. The sections were then incubated overnight with the primary antibody working solution at 4 °C, followed by incubation with the secondary antibody working solution at room temperature in the dark for one hour. After the removal of the working solution and washing, the DAPI working solution was added dropwise and incubation was performed at room temperature in the dark for 10 min, after which the DAPI working solution was removed. An anti-fade mounting agent was applied, and the sections were covered with a coverslip for mounting. Imaging was carried out using a fluorescence microscope (DM2500, Leica, Wetzlar, Germany).

#### 2.2.5. Analysis of Sperm Deformity Rate

In each of the D7 and D42 treatment groups, the tails of the epididymis of three mice were cut into small pieces and incubated in preheated normal saline at 37 °C to fully free the sperm and form sperm suspensions. After brief centrifugation, the supernatant was taken and smeared on slides. For each suspension, three smears were made. A computer-assisted sperm analyzer (SAS Medical, Technology (Beijing) Co., Ltd., Beijing, China) was used to randomly capture 10 non-overlapping fields for each smear, and its integrated morphological analysis software (SAS Computer-Assisted Sperm Analysis System 3.10, CASA) was utilized to automatically identify and count the sperm in the fields. The software is set up to cumulatively analyze at least 200 sperm from each animal sample (i.e., its three smears) and automatically generate preliminary deformity rate data and classify abnormal types. To ensure accuracy, all sperm images marked as abnormal by the software, as well as at least 10% of the normal sperm images randomly selected by the system, are manually rechecked and confirmed. Ultimately, the sperm deformity rate of each animal was calculated based on the data confirmed through manual review.

### 2.3. Statistical Analysis

The experimental data were statistically analyzed using SPSS 23.0 statistical software. The measurement data were expressed as mean ± standard deviation (mean ± SD) after statistical analysis, and the *t*-test was used to compare the differences between the two groups. The differences among multiple groups were analyzed using ANOVA. When the differences were statistically significant and met the homogeneity of variance, the LSD-t test was used for post hoc multiple comparisons; otherwise, Dunnett’s *t*-test was used. A *p*-value < 0.05 was considered statistically significant.

To ensure the objectivity of the experimental results, this study adopted blind methods in the processes of animal grouping, drug preparation, administration, sampling, as well as subsequent index detection and analysis. Except for the final interpretation of the results, the experimenters were unaware of the drugs added to the mice and their grouping.

## 3. Results

### 3.1. Effects of Tet Intervention on the Expression Levels of Marker Molecules in Testicular Tissues of Male Mice Exposed to SiO_2_ (D7 Model)

**Figure 1:**
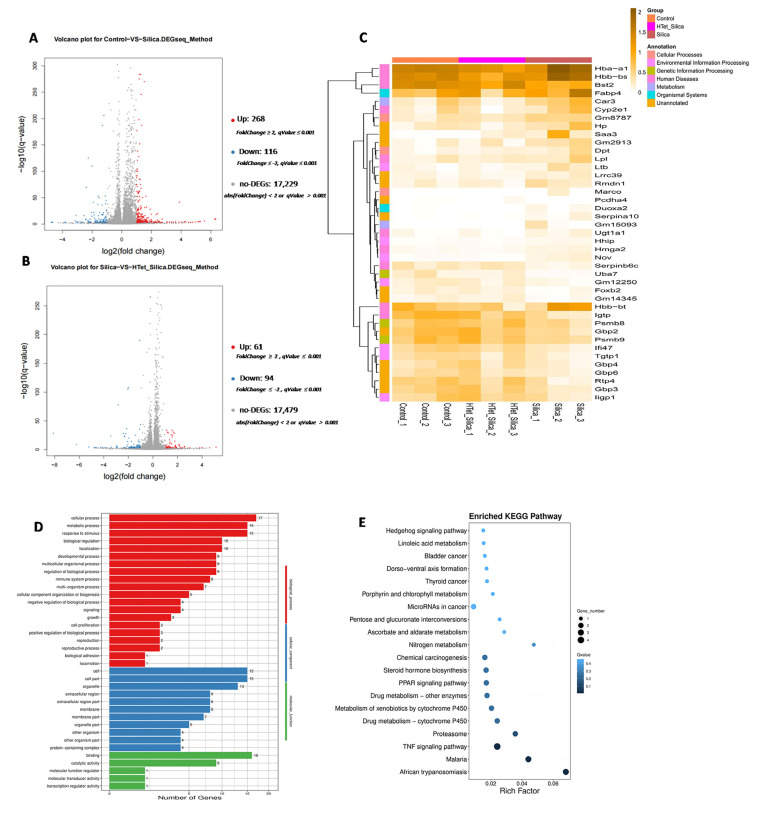

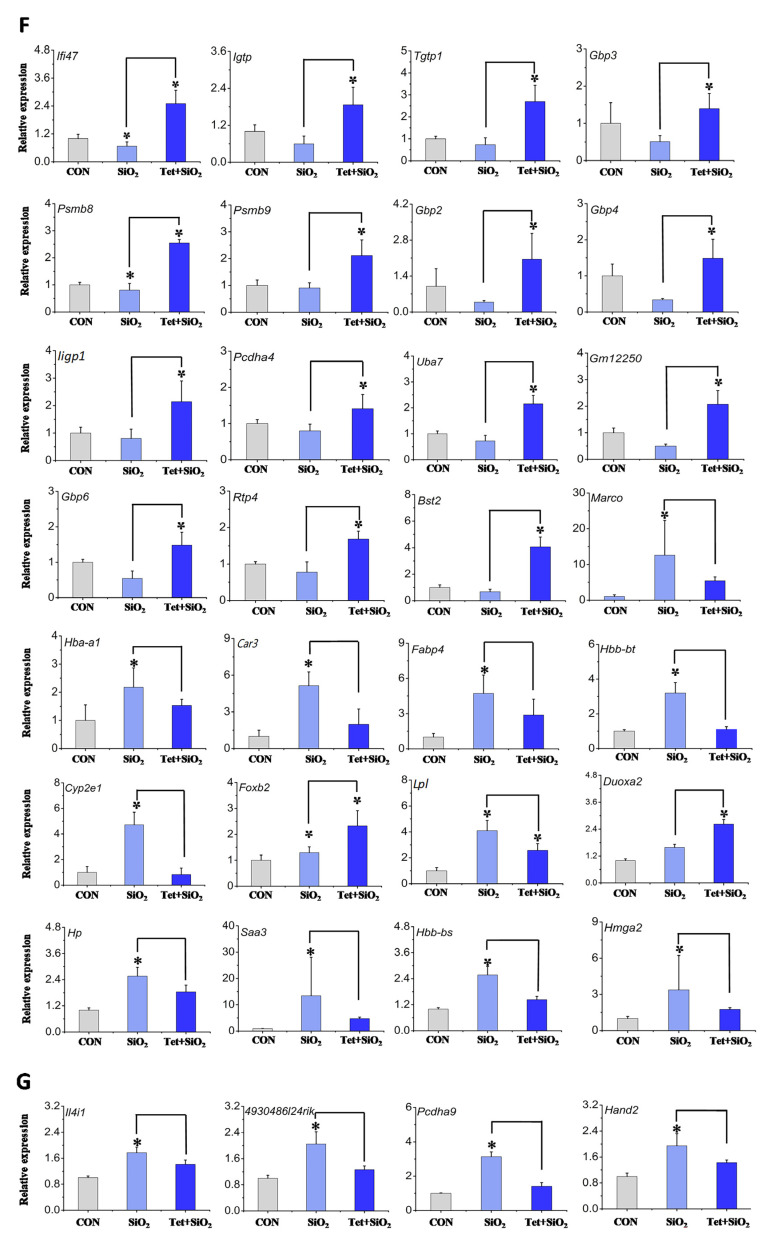

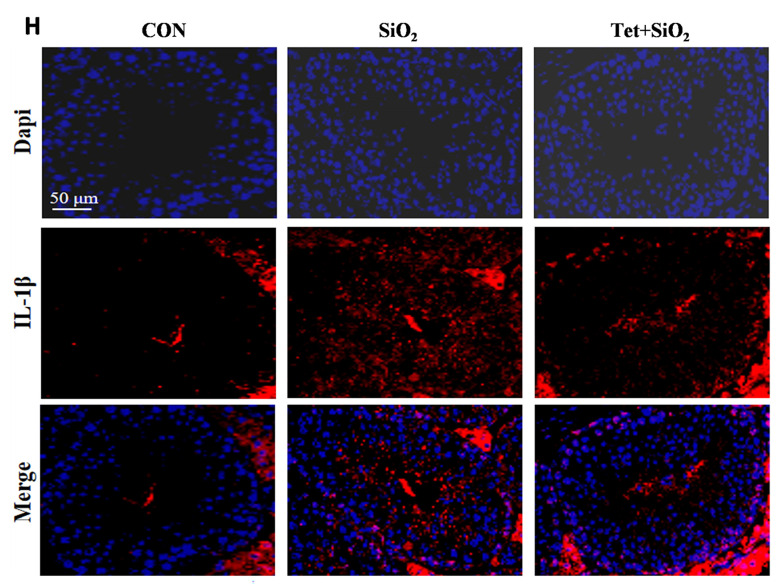
Effect of Tet intervention on the expression level of marker molecules in testicular tissue of male mice exposed to SiO_2_ (D7 model) (**A**): Volcano plot of differentially expressed genes (DEGs) (control group vs. SiO_2_ group; red represents upregulation, blue represents downregulation) (*n* = 3). (**B**): DEGs volcano chart (SiO_2_ group vs. Tet + SiO_2_ group; red represents upward adjustment, blue represents downward adjustment) (*n* = 3). (**C**): Focus on the heat map of DEGs (*n* = 3). (**D**): GO analysis of DEGs (*n* = 3). (**E**): KEGG analysis of DEGs (*n* = 3). (**F**): RT-qPCR verification results (only 28 DEGs consistent with transcriptome sequencing results are shown) (*n* = 3). (**G**): The mRNA expression levels of some key DEGs (*n* = 3). (**H**): In situ expression of IL-1β (IF staining) (*n* = 3). * indicates that *p* < 0.05 compared with the control group. The broken line indicates that *p* < 0.05 compared with the SiO_2_ group in the Tet + SiO_2_ group. In figures (**A**–**G**), *n* = 3 represents three pooled samples, each of which is composed of equal amounts of tissues from three animals.

Based on pool-level expression analysis, the transcriptome sequencing results showed that compared with the control group, 268 upregulated differentially expressed genes (DEGs) and 116 downregulated DEGs were identified in the SiO_2_ group. Compared with the SiO_2_ group, 61 cases of upregulated DEGs and 94 cases of downregulated DEGs were identified in the Tet + SiO_2_ group (Figure 1A,B). To identify DEGs potentially reversed by Tet intervention, we applied an intersection strategy: Up-DEGs (control group vs. SiO_2_ group) ∩ Down-DEGs (SiO_2_ group vs. Tet + SiO_2_ group). For ease of expression, it is defined as the Up–Down model. Down-DEGs (control group vs. SiO_2_ group) ∩ Up-DEGs (SiO_2_ group vs. Tet + SiO_2_ group) is defined as the Down–Up model. This approach yielded 41 key DEGs, comprising 23 in the Up–Down pattern and 18 in the Down–Up pattern (Figure 1C). RT-qPCR validation confirmed the expression trend for 28 (68.29%) of these DEGs (Figure 1F). The core significance of verifying DEGs using RT-qPCR lies in confirming the authenticity, reliability, and quantitative accuracy through an independent “gold-standard” method. Notably, among the key DEGs, we identified four inflammation-associated genes (*Il4i1*, *4930486l24rik*, *Pcdha9*, and *Hand2*) that were also dysregulated in a separate longer-term exposure (D42) model (*p* < 0.05), prompting further investigation. SiO_2_ exposure significantly increased their mRNA levels compared with controls, an effect that was significantly attenuated by Tet co-treatment (Figure 1G). Functional enrichment analysis of these key DEGs in the D7 model revealed that Gene Ontology (GO) terms were predominantly enriched in “response to stimulus” and “metabolic process”, with positive regulation being the dominant mode (Figure 1D). At the KEGG pathway level, significant enrichment was observed in the PPAR signaling pathway and TNF signaling pathway (Figure 1E). This pattern suggests a potential increase in the permeability of the testicular blood–testis barrier and the initiation of an inflammatory stimulus response following SiO_2_ exposure. Consistently, immunofluorescence analysis demonstrated a significant increase in IL-1β intensity upon SiO_2_ exposure (*p* < 0.05), which was also markedly reduced in the Tet + SiO_2_ group (Figure 1H). Collectively, these results indicate that Tet intervention can partially counteract SiO_2_-induced reproductive injury in male mice at the molecular level.

### 3.2. Tet Can Reduce the Rate of Sperm Deformity in the Cauda Epididymidis of Mice Caused by SiO_2_ Exposure (D42 Model)

**Figure 2:**
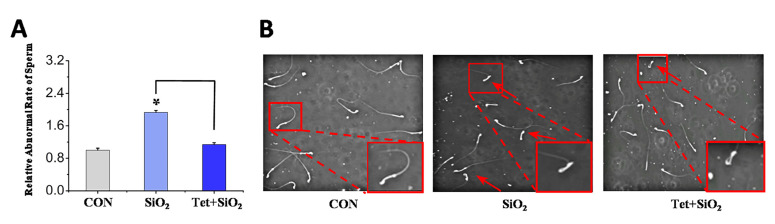
Effect of Tet intervention on the sperm deformity rate in the cauda epididymidis of mice caused by SiO_2_ exposure (D42 model) (**A**): Sperm deformity rate in the cauda epididymidis of mice in each experimental group (*n* = 3). (**B**): Sperm morphology (the red arrow indicates head folding), the red box in the lower right corner is a magnified view of the area within the red box in the upper left corner. * indicates *p* < 0.05 compared with the control group, and the broken line indicates *p* < 0.05 compared with SiO_2_. Scale: 50 μm.

Compared with the control group, the sperm deformity rate in the cauda epididymidis of mice in the SiO_2_ exposure group was significantly increased *(p* < 0.05) (Figure 2A), and the deformity type was mainly “head folding” type (Figure 2B). Compared with the SiO_2_ group, the rate of sperm deformity in the cauda epididymidis of mice in the Tet + SiO_2_ group was significantly reduced (*p* < 0.05) (Figure 2A). It can be concluded from this that Tet can reduce the rate of sperm deformity in the cauda epididymidis of mice caused by SiO_2_ exposure.

### 3.3. The Effect of Tet Intervention on the Expression Level of Marker Molecules in Testicular Tissue of Male Mice Exposed to SiO_2_ (D42 Model)

**Figure 3:**
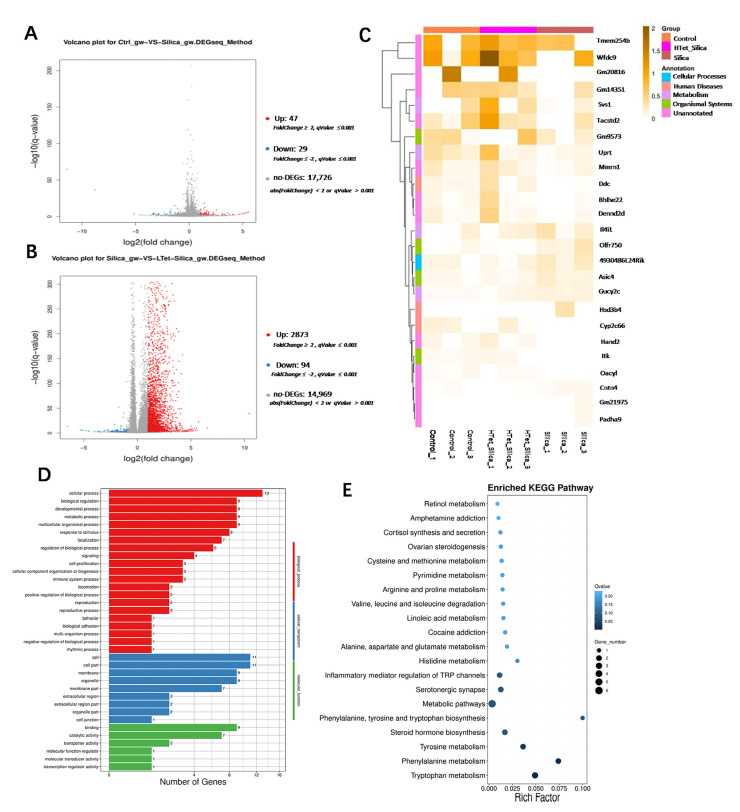

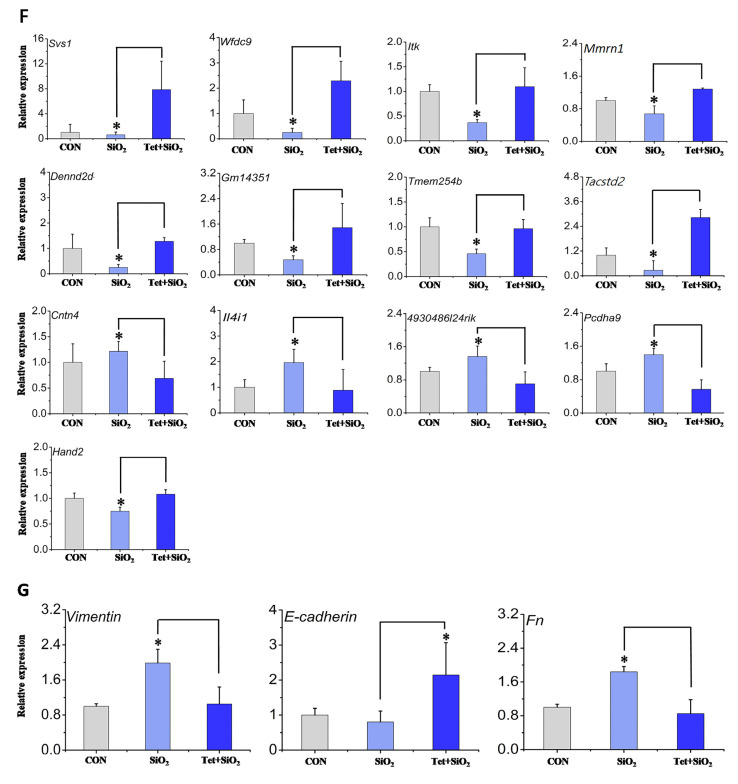

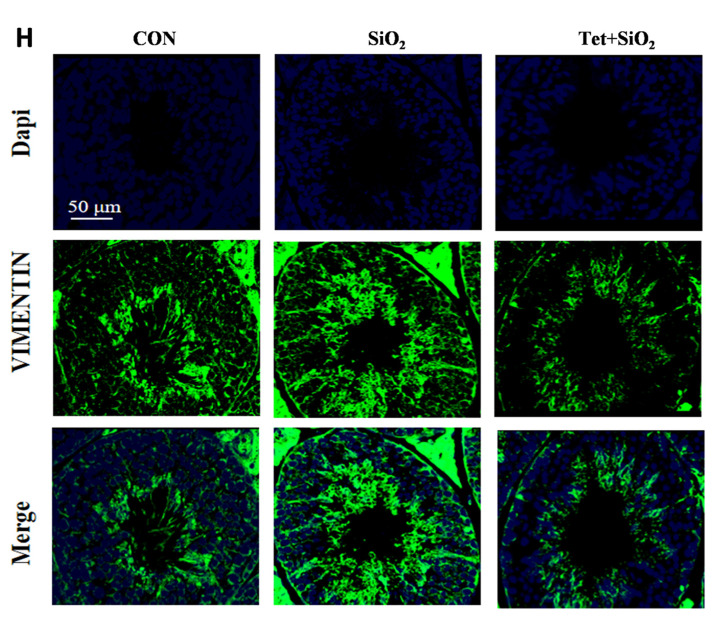
Effects of Tet intervention on the expression levels of marker molecules in the testicular group of male mice exposed to SiO_2_ (D42 model) (**A**): Volcano plot of differentially expressed genes (DEGs) (control group vs. SiO_2_ group; red represents upregulation, blue represents downregulation) (*n* = 3). (**B**): DEGs volcano plot (SiO_2_ group vs. Tet + SiO_2_ group; red represents upward adjustment, blue represents downward adjustment) (*n* = 3). (**C**): Heat maps of DEGs that deserve special attention (*n* = 3). (**D**): GO analysis of DEGs (*n* = 3). (**E**): KEGG analysis of DEGs (*n* = 3). (**F**): RT-qPCR verification results (only show 13 DEGs consistent with transcriptome sequencing results) (*n* = 3). (**G**): mRNA expression levels of fibrosis-related marker molecules (*n* = 3). (**H**): In situ expression of Vimentin (IF staining) (*n* = 3). * indicates that *p* < 0.05 compared with the control group. The broken line indicates that *p* < 0.05 compared with the SiO_2_ group in the Tet + SiO_2_ group. In panels (**A**–**G**), *n* = 3 represents three pooled samples, each of which is composed of equal amounts of tissues from three animals.

Pooled-sample transcriptomic analysis identified 47 upregulated and 29 downregulated DEGs in the SiO_2_ group compared with the control (Figure 3A). In contrast, the Tet + SiO_2_ group exhibited 2873 upregulated and 94 downregulated DEGs relative to the SiO_2_ group alone (Figure 3B). To identify DEGs potentially reversed by Tet intervention in the D42 model, we applied the same intersection strategy as in the D7 analysis: Up-DEGs (control group vs. SiO_2_ group) ∩ Down-DEGs (SiO_2_ group vs. Tet + SiO_2_ group). For ease of expression, this is defined as the Up–Down mode. Down-DEGs (control group vs. SiO_2_ group) ∩ Up-DEGs (SiO_2_ group vs. Tet + SiO_2_ group) is defined as the Down–Up mode. This approach yielded 25 key DEGs, comprising 7 in the Up–Down pattern and 18 in the Down–Up pattern (Figure 3C). RT-qPCR validation confirmed the expression trend for 13 (52%) of these DEGs (Figure 3F). Functional enrichment analysis in the D42 model showed that Gene Ontology (GO) terms remained highly enriched in “developmental process” and “reproduction” (Figure 3D), but with a shift toward negative regulation as the dominant mode. Genes related to cell junction and membrane components continued to be highly represented. KEGG pathway analysis highlighted enrichment in steroid hormone biosynthesis, retinol metabolism, and cysteine and methionine metabolism (Figure 3E). Notably, among these key DEGs, we focused on three fibrosis- and epithelial–mesenchymal transition (EMT)-associated genes: Vimentin, Fibronectin (Fn), and E-cadherin. SiO_2_ exposure significantly upregulated the mRNA levels of Vimentin and Fn while downregulating E-cadherin compared with controls (*p* < 0.05). Tet co-treatment significantly attenuated these changes (*p* < 0.05) (Figure 3G). Consistently, immunofluorescence analysis showed that SiO_2_ exposure significantly increased the fluorescence intensity of Vimentin, an effect that was markedly reduced in the Tet + SiO_2_ group (*p* < 0.05) (Figure 3H). Together, these molecular alterations suggest a progression toward testicular fibrosis and a stagnation of spermatogenesis in the long-term exposure model. Collectively, these results indicate that Tet intervention can partially antagonize the reproductive damage induced by long-term SiO_2_ exposure in male mice. By comparing the key DEGs identified in the D7 (acute) and D42 (chronic) models, we observed a distinct time-dependent shift in the molecular mechanisms underlying SiO_2_-induced reproductive injury.

## 4. Discussion

Silicon dioxide is widely present in nature in both amorphous and crystalline forms [1,2]. Long-term exposure to silica dust, especially when its particle size is less than 10 μm, can induce occupational diseases such as silicosis and occupational tumors, which have a serious impact on human health [6]. At the same time, it also poses a potential threat to other human systems, such as the reproductive system. Silica exposure in the occupational environment mainly affects occupational groups such as coal miners and stone miners, the vast majority of whom are male and of childbearing age (in the field of public health, 15–49 years old is often used as the reference range for men of childbearing age). Therefore, we focus on the secondary male reproductive damage effect after lung injury caused by silica exposure. The results of bibliometric analysis show that few studies have focused on this field. Tet has been used in the treatment of silicosis due to its definite anti-inflammatory and anti-fibrotic properties [19]. Some studies have found that Tet can improve male erectile dysfunction [17], suggesting that Tet has a potential protective effect on male reproductive function. In conclusion, this study is dedicated to resolving the scientific issue of male reproductive damage caused by silica exposure, and at the same time exploring the antagonistic effect of Tet on male reproductive damage caused by silica.

Given that this study focuses on the secondary male reproductive damage effect after lung injury caused by silica exposure, the modeling methods from previous studies and the previous research of our research group were adopted. Two key time points should be focused on: D7, the male reproductive injury model corresponding to the pulmonary inflammation stage, and D42, the male reproductive injury model corresponding to the pulmonary fibrosis stage. The research results show that in the D7 model, the mRNA expression levels of inflammation-related genes *Il4iI* and IL-1β in the testicular tissues of mice exposed to silica alone are significantly increased. Here, we only take these two indicators as examples. In fact, the expression levels of other inflammation-related genes have also been affected to varying degrees. Studies have shown that IL-1β has a pro-inflammatory effect and exists in small amounts in the testicles of normal men. Significantly, when the level of IL-1β increases, it can lead to testicular inflammation and spermatogenic disorders, affecting male fertility [20], and a similar phenomenon occurred in this study. Therefore, it is suggested that exposure to silica dust can cause inflammatory damage to the testicles of mice in a short period of time. Studies have shown that SiNPs can enter the bloodstream through the respiratory system and reach testicular tissue via the blood–testis barrier [21]. This study, through transcriptome analysis, found that under SiO_2_ exposure, the permeability of the blood–testis barrier increases, making it possible for SiO_2_ to reach testicular tissue. When SiO_2_ enters the body, the level of IL-1β in the testis increases, suggesting that the NLRP3 inflammasome pathway is activated [22], which is highly consistent with the enrichment results of pro-inflammatory pathways such as the TNF signaling pathway in our transcriptome data. This suggests that SiO_2_ may disrupt the blood–testis barrier through NLRP3/IL-1β, causing testicular inflammation. More importantly, in the D7 model, it was found that the level of IL-1β in the testicles of mice significantly decreased after exposure to Tet combined with silica. This phenomenon also existed in the study by Ren et al., who found that the level of IL-1β in male mice was significantly reduced after treating them with different doses of Tet for two months [23]. Studies have shown that Tet can alleviate the damage caused by SiO_2_ by inhibiting the NLRP3 inflammasome pathway. All of the above suggest that Tet may be able to inhibit the activation of the NLRP3 inflammasome pathway induced by SiO_2_, thereby reducing the level of IL-1β [24]. The D7 model suggests that Tet can ameliorate the testicular inflammatory damage caused by silica dust in mice to a certain extent.

In the D42 model, we observed in testicular tissue that the expressions of Vimentin and FN significantly increased and the expression of E-cadherin significantly decreased after silica exposure, which was consistent with the changes in the key molecular characteristics of epithelial–mesenchymal transition (EMT) [25] and similar alterations were also observed in the study by Ma [26]. Vimentin, Fn, and E-cadherin are some of the markers of EMT events and are closely related to organ fibrosis. EMT events continue to occur when the inflammatory response persists, eventually leading to the fibrosis of tissues and organs [27]. This suggests that a related pathological process similar to EMT may have been activated in testicular tissue. After combined exposure to Tet, it was observed that the expression levels of *Vimentin* and *Fn* were decreased significantly, while the expression level of *E-cadherin* was increased significantly. This change was reversed after combined exposure to Tet. It was observed that the expressions of Vimentin and FN decreased significantly, and the expression of E-cadherin increased significantly, which was consistent with the reported inhibition of EMT by Tet in other tissues [28,29]. Meanwhile, in this model, the deformity rate of mouse sperm significantly increased only under silica conditions, mainly characterized by “head folding”. Under Tet intervention, it was found that the sperm deformity rate was greatly reduced. This phenomenon also exists in Azenabor’s study [5], which may be related to the fact that a large amount of inflammatory factors are released during testicular injury, causing damage to sperm, while Tet can inhibit the inflammatory factors and reduce the damage to sperm. Based on the above findings, we speculate that after silica exposure, EMT-like pathological changes may be highly associated with testicular damage and elevated sperm deformity in mice, ultimately causing damage to the reproductive capacity of mice. It is gratifying that Tet can alleviate the above-mentioned male reproductive damage caused by silica exposure.

The equivalent dose (6.5 mg/kg) in this study was higher than the systemic occupational exposure load (1.3–3.9 mg/kg), indicating that the experiment simulated the potential toxic endpoints of long-term occupational risks through high-dose short-term exposure, providing a mechanistic warning for the reproductive toxicity of SiO_2_ [30,31]. The research results can provide new ideas for the protection of occupational exposure to silica. Low-dose Tet can offer protection against the reproductive health of high-risk silica populations. A very important point is that even with long-term use at this dose, there is no obvious liver or kidney toxicity to the body.

Although this study has shed light on the mechanism by which Tet antagonizes male reproductive damage caused by silica, due to its short research duration and the fact that the study only focused on the transcriptome, it still needs to be combined with epidemiological studies and systematic safety evaluations before it can be applied in clinical practice. In this study, intranasal instillation was adopted as the exposure model. Its advantages lie in its controllable operation and the ease of inducing local high-concentration effects to explore the mechanism. However, it cannot fully simulate the uniform distribution and deposition dynamics of aerosols in the lungs under natural inhalation exposure. Therefore, the conclusion of this study provides important mechanism clues for understanding how Tet antagonizes male reproductive damage caused by silica. However, its universality in real inhalation exposure scenarios awaits direct verification using standard inhalation exposure equipment in the future. The sample merging strategy adopted in this study, although effectively revealing the transcriptome variation trend at the group level, due to the fact that the analysis unit was an RNA pool and the number of pools was limited (*n* = 3 per group), yielded results that could not infer individual variations and had limited statistical power. Future research needs to provide verification at the individual animal level to confirm the robustness of these findings and explore individual differences [32,33,34,35]. This study only evaluated a limited number of EMT-related markers. Although the changes in Vimentin, Fibronectin, and E-cadherin suggest EMT-like alterations, to confirm that the complete EMT process is regulated, future research requires the detection of core transcription factors (such as Snail and Twist) and supplementation with cell morphology and functional analysis [36]. Furthermore, micrometer-sized silica is always present. Nano-sized silica with smaller particle sizes has long been a resident of our occupational and living environments. Considering that nano-sized silica particles can penetrate the blood–testis barrier and accumulate in testicular tissue, they can cause reproductive toxicity [9,10,11]. Next, the research ideas and technical methods of systems toxicology should be adopted to explore the reproductive toxicity caused by nanoscale silica particles.

## 5. Conclusions

Tet can alleviate the reproductive damage caused by silica exposure in male mice to a certain extent. The results of this study not only expand our understanding of the bodily damage caused by silica exposure in occupational environments, but also provide new ideas for the clinical treatment of male reproductive damage caused by silica exposure with Tet.

## Figures and Tables

**Table 1:** Information on the main reagents used in this study.

Reagent	Manufacturer	Product Number
FastKing gDNA Dismising RT SuperMix	TIANGEN Biotech (Beijing) Co., Ltd., Beijing, China	KR118
2X Universal SYBR Green Fast qPCR Mix	ABclonal Technology Co., Ltd., Wuhan, China	RK21203
RNAsimple Total RNA Kit	TIANGEN Biotech (Beijing) Co., Ltd., Beijing, China	DP419
Goat Serum	Beyotime Biotechnology Co., Ltd., Shanghai, China	C0265
PBS	Servicebio Technology (Wuhan) Co., Ltd., Wuhan, China	G4202
Rabbit polyclonal antibody to IL1 beta	Affinity Biosciences (Jiangsu) Co., Ltd., Zhenjiang, China	AF5103
Rabbit polyclonal anti-Fibronectin (Fn) antibody	Affinity Biosciences (Jiangsu) Co., Ltd., China	AF5335
Rabbit polyclonal antibody to Vimentin	Affinity Biosciences (Jiangsu) Co., Ltd., China	AF7013
Rabbit polyclonal antibody to E-cadherin	Affinity Biosciences (Jiangsu) Co., Ltd., China	AF0131
Universal antibody dilution buffer	SEVEN Bioteon (Shenzhen) Co., Ltd., Shenzhen, China	SW161-02
Anti-fluorescence quenching tablets (containing DAPI)	Shandong Sparkjade Biotechnology (Jinan) Co., Ltd., Jinan, China	EE0015

**Table 2:** Primer-related information used in this study (D7 model).

Gene	Accession No.	Forward Primer	Reverse Primer	Length	Annealing Temperature (°C)
*Ifi47*	NM_008330.2	TCTCCAGAAACCCTCACTGGT	TCAGCGGATTCATCTGCTTCG	200	60
*Igtp*	NM_018738.4	CTCATCAGCCCGTGGTCTAAA	CACCGCCTTACCAATATCTTCAA	102	60
*Tgtp1*	NM_011579.3	TGCACAGATGGGGATGAATTTC	TCACTGTCGAGAGACTCCTGA	159	60
*Gbp3*	NM_018734.4	GAGGCACCCATTTGTCTGGT	CCGTCCTGCAAGACGATTCA	162	60
*Psmb8*	NM_010724.2	ATGGCGTTACTGGATCTGTGC	CGCGGAGAAACTGTAGTGTCC	111	60
*Psmb9*	NM_013585.3	CATGAACCGAGATGGCTCTAGT	TCATCGTAGAATTTTGGCAGCTC	111	60
*Gbp2*	NM_010260.1	CTGCACTATGTGACGGAGCTA	GAGTCCACACAAAGGTTGGAAA	115	60
*Gbp4*	NM_008620.4	GGAGAAGCTAACGAAGGAACAA	TTCCACAAGGGAATCACCATTTT	136	60
*Iigp1*	NM_001146275.1	CATCCCTTCTCTGACCTTTCTCTTG	GCCTCCACCTGATCCACCTC	147	60
*Pcdha4*	NM_007766.3	CCCCAGTTTATCTGATTCAAGGG	GCTGTTGCTGTTGACACCG	271	60
*Uba7*	NM_023738.4	CTACGAGCGACTCCATATACCT	TACACACAGGGTAGGGAGCAT	280	60
*Gm12250*	NM_001135115.1	GTTCGGACCAAAATTGACAGTG	ACACGAGTAGAGGCTGCGTTA	140	60
*Gbp6*	NM_194336.2	GTTCCAGGAAGTAACAAAGGCT	ATCCCTAGTCTATTCCCAGTGAC	102	60
*Rtp4*	NM_023386.6	TGGGAGCAGACATTTCAAGAAC	ACCTGAGCAGAGGTCCAACTT	179	60
*Bst2*	NM_198095.3	TGTTCGGGGTTACCTTAGTCA	GCAGGAGTTTGCCTGTGTCT	179	60
*Marco*	NM_010766.3	ACAGAGCCGATTTTGACCAAG	CAGCAGTGCAGTACCTGCC	149	60
*Hba-a1*	NM_008218.2	CACCACCAAGACCTACTTTCC	CAGTGGCTCAGGAGCTTGA	201	60
*Car3*	NM_007606.3	GGCGAGTTCCAGATTCTTCTTGATG	GTGGTGAAGGAGCCGTGATAGG	137	60
*Fabp4*	NM_024406.4	AAGGTGAAGAGCATCATAACCCT	TCACGCCTTTCATAACACATTCC	133	60
*Hbb-bt*	NM_008220.5	GCCGATGAAGTTGGTGGTGAG	ATGATAGCAGAGGCAGAGGATAGG	107	60
*Cyp2e1*	NM_021282.3	CGTTGCCTTGCTTGTCTGGA	AAGAAAGGAATTGGGAAAGGTCC	105	60
*Foxb2*	NM_008023.2	TTCCTACAGCGACCAAAAGCC	CCGAGGGATCTTGATGAAACAG	208	60
*Lpl*	NM_008509.2	CAACAAGGTCAGAGCCAAGAGAAG	GTTGCTTGCCATCCTCAGTCC	122	60
*Duoxa2*	NM_025777.3	GACGGGGTGCTACCCTTTTAC	CCCACGGATTCCAGGCAAG	129	60
*Hp*	NM_017370.2	GCTATGTGGAGCACTTGGTTC	CACCCATTGCTTCTCGTCGTT	101	60
*Saa3*	NM_011315.3	TGCCATCATTCTTTGCATCTTGA	CCGTGAACTTCTGAACAGCCT	248	60
*Hbb-bs*	NM_001201391.1	GCCGATGAAGTTGGTGGTGAG	ATGATAGCAGAGGCAGAGGATAGG	107	60
*Hmga2*	NM_010441.3	GAGCCCTCTCCTAAGAGACCC	TTGGCCGTTTTTCTCCAATGG	106	60
*Il4i1*	NM_010215.3	CTGCCCAAGAGAGCTGAAGACA	ACCACTACCACCTTCTGGGG	231	60
*4930486l24rik*	NM_178098.2	ATGATCGCTGTTCTCTTCCTAGC	GGTATTCCCAATTATGCAGCTCA	199	60
*Pcdha9*	NM_138661.1	GAATTTACGGGATCGGTTTCTCT	TGAGTCAGTAGCATTCAGCCAT	147	60
*Hand2*	NM_010402.4	GCAGGACTCAGAGCATCAACA	AGGTAGGCGATGTATCTGGTG	124	60

**Table 3:** Primer-related information used in this study (D42 model).

Gene	Accession No.	Forward Primer	Reverse Primer	Length	Annealing Temperature (°C)
*Svs1*	NM_172888.3	GGTAGGAAGGACCTTGGTTCT	CCTCACCACTCAAGTCCCAC	125	60
*Wfdc9*	NM_001160414.2	CGTCCTCACTGTATCTGCCCATG	TCACGCACTTCCGCACCTTC	149	60
*Itk*	NM_010583.3	GGAAGAAGCGCACGTTGAAG	ATGCACGACCTGAAAAGGGTA	116	60
*Mmrn1*	NM_001163507.1	GGTCTTCAGGCTTACCAACAC	GAGTGGCCGAGAGCACTTG	128	60
*Dennd2d*	NM_028110.2	GCTGCTCCGAAATCGCTTG	CTAGATGGAGAATGCTCCTGGA	105	60
*Gm14351*	NM_001085552.2	GAGCCACAGCCAGAAGCCATAG	CTTGTTGCCAACTCCTCCAAACTG	89	60
*Tmem254b*	NM_026679.2	GGCAGCTTGTTCTGGTTCAC	GGCTCTGATAAGGGATGCTCTG	91	60
*Tacstd2*	NM_020047.3	ACAACGATGGCCTCTACGAC	TTTGGTCTCCCTTGTCCGTG	129	60
*Cntn4*	NM_001411734.1	GGACATTGTGTTTACGTGGACA	CAGTTGGATGTTTCGGATCATCA	124	60
*Vimentin*	NM_011701.4	CGTCCACACGCACCTACAG	GGGGGATGAGGAATAGAGGCT	74	60
*Fn*	NM_010233.2	GCTCAGCAAATCGTGCAGC	CTAGGTAGGTCCGTTCCCACT	115	60
*E-cadherin*	NM_009864.3	CAGGTCTCCTCATGGCTTTGC	CTTCCGAAAAGAAGGCTGTCC	175	60

## Data Availability

The original contributions presented in this study are included in the article/Supplementary Materials. Further inquiries can be directed to the corresponding authors.

## Supplementary Materials

The following supporting information can be downloaded at: https://www.mdpi.com/article/10.3390/toxics14010087/s1, Figure S1: Scatter plot of RNA-seq vs. RT-qPCR log_2_FC (D7 Con vs. SiO_2_); Figure S2: Scatter plot of RNA-seq vs. RT-qPCR log_2_FC (D7 SiO_2_ vs. Tet + SiO_2_); Figure S3: Scatter plot of RNA-seq vs. RT-qPCR log_2_FC (D42 Con vs. SiO_2_); Figure S4: Scatter plot of RNA-seq vs. RT-qPCR log_2_FC (D42 SiO_2_ vs. Tet + SiO_2_); Table S1: Comparison of expression changes between RNA-seq and RT-qPCR for validated genes (D7 model); Table S2: Comparison of expression changes between RNA-seq and RT-qPCR for validated genes (D42 model).
