# Supplementary material for: The Protective Effect and Molecular Mechanism of Tetrandrine on Male Reproductive Damage Caused by Silicon Dioxide
Source: Toxics. 2026 Jan 18;14(1):87. doi: 10.3390/toxics14010087 (PMC12846249; doi:10.3390/toxics14010087)
Supplement: Supplementary file 1 [file toxics-14-00087-s001.zip › toxics-4023283-supplementary.pdf]

1. The complete experimental process and calculation methods for transcriptome sequencing and bioinformatics analysis

This study adopted the following complete transcriptome sequencing and analysis process: Stron-specific libraries of total RNA in qualified testicular tissues were constructed using the NEBNext® Ultra™ RNA Library Prep Kit, and double-ended 150 bp sequencing was performed on the Illumina NovaSeq platform. On average, each sample obtained approximately 48.9 million original reads. After the original data was subjected to FastQC quality control, low-quality, linker, and high-N base sequences were filtered using SOAPnuke (v1.5.2, parameters: -l 15-q 0.2-n 0.1-Q 2) to obtain high-quality Clean Reads. Subsequently, Clean Reads were aligned to the mouse reference genome UCSC mm10 (GRCm38) using HISAT2 (v2.2.1) (download address: ftp://hgdownload.cse.ucsc.edu/goldenPath/mm10) and featureCounts quantitative (v2.0.1) was used for gene expression. Differential expression analysis was performed using DESeq2 (v1.34.0), with the screening criteria being log2FC > 1 and FDR < 0.05. Finally, clusterProfiler was used to conduct GO and KEGG enrichment analyses on significantly differentially expressed genes.

2. Comparison of expression changes between RNA-seq and RT-qPCR for validated genes

Table S1. Comparison of expression changes between RNA-seq and RT-qPCR for validated genes (D7 model).

Gene	Group	RNA-seq log_2FC	RT-qPCR log_2FC
Hba-a1	Con vs SiO_2	1.18	1.07
	SiO_2 vs Tet+SiO_2	-1.93	-0.49
Hbb-bs	Con vs SiO_2	1.12	1.38
	SiO_2 vs Tet+SiO_2	-1.98	-0.89
Bst2	Con vs SiO_2	-1.53	-0.51
	SiO_2 vs Tet+SiO_2	1.30	2.00
Fabp4	Con vs SiO_2	1.66	2.26
	SiO_2 vs Tet+SiO_2	-1.32	-0.78
Car3	Con vs SiO_2	1.35	2.32
	SiO_2 vs Tet+SiO_2	-2.11	-1.56

Cyp2e1	Con vs SiO_2	1.41	2.26
	SiO_2 vs Tet+SiO_2	-2.32	-2.58
Hp	Con vs SiO_2	1.67	1.32
	SiO_2 vs Tet+SiO_2	-1.96	-0.47
Saa3	Con vs SiO_2	6.19	3.64
	SiO_2 vs Tet+SiO_2	-2.91	-1.32
Lpl	Con vs SiO_2	1.31	2.00
	SiO_2 vs Tet+SiO_2	-1.08	-0.68
Marco	Con vs SiO_2	5.17	3.58
	SiO_2 vs Tet+SiO_2	-4.09	-1.00
Pcdha4	Con vs SiO_2	-4.52	-0.32
	SiO_2 vs Tet+SiO_2	4.99	0.81
Duoxa2	Con vs SiO_2	3.75	0.68
	SiO_2 vs Tet+SiO_2	-4.59	0.75
Hmga2	Con vs SiO_2	1.02	1.72
	SiO_2 vs Tet+SiO_2	-1.29	-0.87
Uba7	Con vs SiO_2	-2.59	-0.74
	SiO_2 vs Tet+SiO_2	1.84	1.74
Gm12250	Con vs SiO_2	-2.59	-1.00
	SiO_2 vs Tet+SiO_2	1.84	2.00
Foxb2	Con vs SiO_2	-1.77	0.26
	SiO_2 vs Tet+SiO_2	1.82	0.87
Hbb-bt	Con vs SiO_2	1.10	1.68
	SiO_2 vs Tet+SiO_2	-2.25	-1.54
Igtp	Con vs SiO_2	-2.38	-0.74
	SiO_2 vs Tet+SiO_2	1.77	1.74

Psmb8	Con vs SiO_2	-1.07	-0.32
	SiO_2 vs Tet+SiO_2	1.08	1.64
Gbp2	Con vs SiO_2	-1.59	-1.51
	SiO_2 vs Tet+SiO_2	1.60	2.58
Psmb9	Con vs SiO_2	-1.26	-0.15
	SiO_2 vs Tet+SiO_2	1.32	0.64
Ifi47	Con vs SiO_2	-1.84	-0.51
	SiO_2 vs Tet+SiO_2	1.55	1.84
Tgtp1	Con vs SiO_2	-1.57	-0.51
	SiO_2 vs Tet+SiO_2	1.41	1.89
Gbp4	Con vs SiO_2	-1.28	-2.00
	SiO_2 vs Tet+SiO_2	1.69	2.58
Gbp6	Con vs SiO_2	-1.36	-1.00
	SiO_2 vs Tet+SiO_2	1.41	1.58
Rtp4	Con vs SiO_2	-1.99	-0.51
	SiO_2 vs Tet+SiO_2	1.82	1.89
Gbp3	Con vs SiO_2	-1.47	-1.00
	SiO_2 vs Tet+SiO_2	1.64	1.49
Iigp1	Con vs SiO_2	-1.20	-0.32
	SiO_2 vs Tet+SiO_2	1.51	1.32

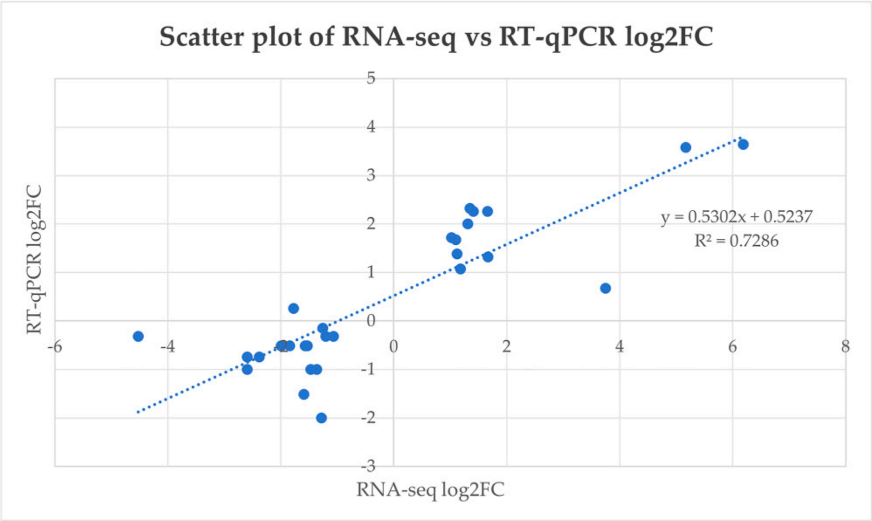

Figure S1 Scatter plot of RNA-seq vs RT-qPCR log_2FC (D7 Con vs SiO_2).

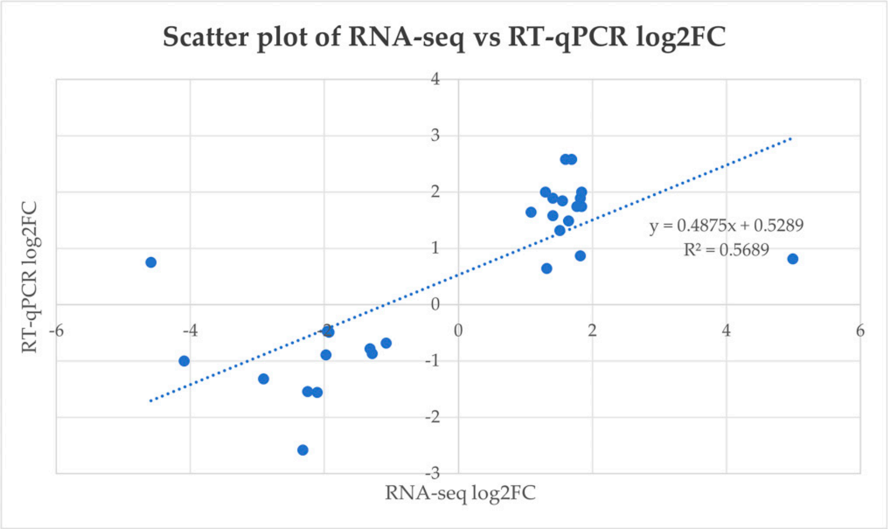

Figure S2 Scatter plot of RNA-seq vs RT-qPCR log_2FC (D7 SiO_2 vs Tet+SiO_2).

Table S2. Comparison of expression changes between RNA-seq and RT-qPCR for validated genes (D42 model).

Gene	Group	RNA-seq log_2FC	RT-qPCR log_2FC
Tmem254b	Con vs SiO_2	-1.09	-1.32
	SiO_2 vs Tet+SiO_2	1.76	1.17
Wfdc9	Con vs SiO_2	-1.31	-1.73
	SiO_2 vs Tet+SiO_2	3.86	2.87

Gm14351	Con vs SiO_2	-1.73	-0.74
	SiO_2 vs Tet+SiO_2	1.85	1.22
Svs1	Con vs SiO_2	-1.23	-0.51
	SiO_2 vs Tet+SiO_2	3.76	3.51
Tacstd2	Con vs SiO_2	-1.67	-1.74
	SiO_2 vs Tet+SiO_2	3.58	3.70
Mmrn1	Con vs SiO_2	-1.09	-0.51
	SiO_2 vs Tet+SiO_2	1.79	0.89
Dennd2d	Con vs SiO_2	-1.68	-1.74
	SiO_2 vs Tet+SiO_2	3.56	2.12
Il4i1	Con vs SiO_2	1.84	0.93
	SiO_2 vs Tet+SiO_2	-1.05	-1.08
4930486L24Rik	Con vs SiO_2	1.67	0.38
	SiO_2 vs Tet+SiO_2	-1.44	-0.89
Hand2	Con vs SiO_2	-3.33	-0.42
	SiO_2 vs Tet+SiO_2	3.74	0.55
Itk	Con vs SiO_2	-2.85	-0.74
	SiO_2 vs Tet+SiO_2	2.42	0.87
Cntn4	Con vs SiO_2	1.79	0.26
	SiO_2 vs Tet+SiO_2	-1.86	-1.00
Pcdha9	Con vs SiO_2	5.23	0.38
	SiO_2 vs Tet+SiO_2	-5.13	-1.38

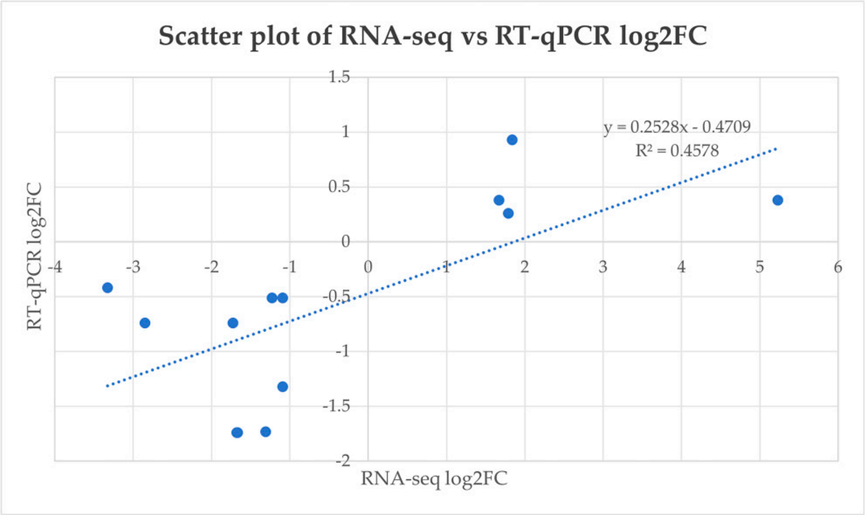

Figure S3 Scatter plot of RNA-seq vs RT-qPCR log_2FC (D42 Con vs SiO_2).

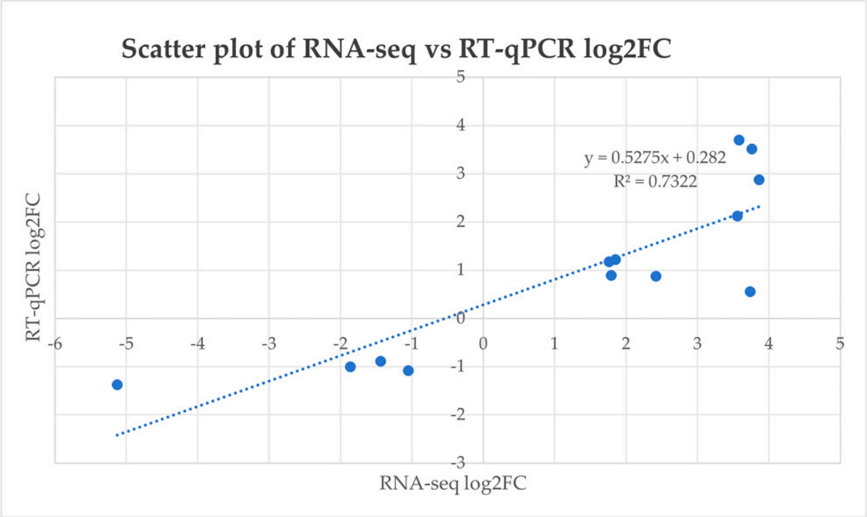

Figure S4 Scatter plot of RNA-seq vs RT-qPCR log_2FC (D42 SiO_2vs Tet+SiO_2).
